# Evaluating the impact of an after-school program on overhand throwing and perceived motor competence among students from low socio-economic backgrounds in the U.S.

**DOI:** 10.3389/fspor.2025.1500723

**Published:** 2025-01-27

**Authors:** Omar Albaloul, Kahyun Nam, Pamela Hodges Kulinna, Conner Acri

**Affiliations:** ^1^ Mary Lou Fulton Teachers College, Arizona State University, Mesa, AZ, United States; ^2^ Kuwait University, Kuwait City, Kuwait; ^3^ University of Minnesota, Duluth, MN, United States

**Keywords:** motor skill, physical activity, physical education, PETE, CSPAP, physical literacy

## Abstract

The study examined the effect of a 5-week Comprehensive School Physical Activity Program (CSPAP) on overhand throw skills and perceived motor competence in students from low socio-economic backgrounds. Participants were children recruited from a Title 1 school in the Southwest U.S. Students were assigned to either CSPAP condition (*n* = 60, 26 boys, 34 girls; Mgrade = 4.27, SD = .43) or a control condition (*n* = 20, 7 boys, 13 girls; Mgrade = 4.00, SD = .35). Overhand throwing and perceived motor competence were assessed at baseline and post-intervention using the Test of Gross Motor Development—2nd Edition and Perceived Motor Competence for Children (PMC-C). The Analysis of Covariance test showed that students who participated in the CSPAP performed statistically significant improvements in the overhand throw compared to control students in the post-test *p* < .001, with a moderate effect size. However, there was no significant difference in the PMC-C score after the 5-week intervention between the CSPAP students and control students. CSPAP can improve overhand throwing in students from low-income families, but new strategies are needed to enhance perceived motor competence in after-school contexts.

## 1 Introduction

The prevalence of physical inactivity among children and youth in the United States is alarmingly high. Approximately 75% of this population fails to meet the 2018 Physical Activity Guidelines for Americans, which recommend a minimum of 60 min of moderate-to-vigorous physical activity (MVPA) per day (1), and are displaying sub-optimal standards for cardiorespiratory fitness (2). Barnett et al. (3) expressed concern that many children today may live more sedentary compared to those of past generations due to increased television use and a lack of access to safe, outdoor play areas. These changes have led to a significant decrease in motivation and opportunities for children to engage in physical activities (PAs) that involve running, jumping, and body movement (4).

While participating in PA is crucial in preventing and reducing obesity and overweight as well as low cardiorespiratory fitness among young children (5), approximately 75% of children in the U.S. fail to meet the established 60 min per day of MVPA recommended by the Centers for Disease Control (6).

This issue is particularly evident among children from low socioeconomic backgrounds, who tend to be less physically active than their peers from medium- and high-socioeconomic status groups (7, 8). Factors contributing to this disparity include limited physical activity (PA) opportunities in their neighborhoods, safety concerns (9), lack of supervision (10), and reduced access to alternative PA options (11). As a result, these children face a higher risk of adopting sedentary lifestyles (12, 13). Addressing these challenges and promoting increased PA can play a crucial role in breaking the cycle of inactivity. Notably, research shows that more active children tend to perceive themselves as more competent in motor skills, which is linked to better development of these skills (14).

### 1.1 Gross motor skills and children with low socioeconomic status

Gross motor skills are foundational elements of PA and include locomotor (e.g., broad jump, gallop, slide), object-control (e.g., throw, stationary dribble, kick), and stability skills (e.g., single leg stance) (15). Gross motor skills involve coordinating major body parts like arms and legs for activities such as jogging, kicking, and throwing (16). Since these skills utilize major body parts and movements, developing them is crucial for core stabilization, proprioception, and body control (17).

Gross motor skills are crucial for acquiring more advanced skills necessary for activities like sports, dance, and recreational PA, and are integral to the Physical Education curricula at the elementary school level (18–20). A lack of development of these skills at an early age often leads to failure to master these skills in adulthood (21). SHAPE America (22) emphasizes in Standard 1 the importance of developing competency in a variety of motor skills and movement patterns as a foundation for physical literacy.

Children's motor skill acquisition can predict adult PA patterns. In a 20-year longitudinal study, Lloyd et al. (23) examined the long-term association between motor skills at age 6 and PA at age 26. They found that proficient motor skills at age 6 were strongly associated with greater engagement in leisure PAs at age 26.

While it is critical for children to develop these skills, many cannot achieve competency in gross motor skills (24). Children from lower social classes tend to have lower gross motor skill development levels than those from middle and high social classes (25, 26, 62). Their limited access to PA and sports participation outside of school settings hinders their development of gross motor skills (27). Children with low gross motor skills may also have low levels of perceived competence for PA (28).

### 1.2 Perceived motor competence and children with low-socioeconomic backgrounds

Perceived motor competence (PMC-C) is a psychological mental construct that falls within the sub-domain of physical competence, specifically referring to an individual's self-assessment of their actual motor competence (29). An individual's self-perception of motor skills can be a significant intermediary factor between motor skills and PA during childhood (30). PMC-C serves as the foundation for one's emotions, motivations, and behavior (31). It is known to play both a critical and determinant role in promoting PA among children and adolescents (20).

PMC-C has been consistently identified as a significant correlate of an individual's actual motor competence (32, 33). On the other hand, the apprehension that comes with failing the execution of gross motor actions can represent a barrier to sustained PA engagement over the course of one's life (34). A child's self-perception of their competence level, regardless of its accuracy, can have implications for their PA, emotional and social well-being (35, 63). Given that children are likely to withdraw from activities when they do not feel competent (20), PMC-C could serve as a critical predictor of PA levels. Children's perception of their capabilities to perform gross motor skills can mediate the relationship between actual motor competence and PA, suggesting that children's PMC-C significantly influences their PA engagement (33, 36, 37).

The impact of PMC-C on PA has also been studied qualitatively. Whitehead and Biddle (38) interviewed 47 adolescents in the United Kingdom to investigate the perceptions of PA among adolescent girls. The participants were asked about the factors related to PA and why they were perceived as influential in their PA. The study found that less active adolescents were concerned about their skills and how people perceived their skills, which influenced their willingness to participate in PA, regardless of their actual skill level. This shows that perceptions of competence and social evaluation play a critical role in determining the level of PA engagement.

Gross motor skills and PMC-C are linked to higher levels of PA. Developing gross motor skills enhances children's ability to participate in various PAs, and higher PMC-C boosts their confidence, making them more likely to engage in and enjoy PA (29, 36). Given this association, interventions aimed at improving these skills are important, especially since children from lower socioeconomic backgrounds are often less physically active compared to their peers from middle and high-economic backgrounds.

A Comprehensive School Physical Activity Program (CSPAP) consists of five key components designed to expand PA opportunities across the entire school community, particularly focusing on students. These components are: (a) quality Physical Education, (b) before- and after-school programs, (c) PA during school hours (such as classroom PA breaks and recess), (d) staff involvement, and (e) family and community engagement (39). The primary goal of CSPAP is to increase PA opportunities before, during, and after the school day. However, as Burns et al. (40) suggest, CSPAP may also contribute to the development of children's gross motor skills by providing more opportunities for physical activity.

Researchers have extensively examined the impact of CSPAP and found it to improve motor skills (41–45). Despite this potential benefit, only two studies have specifically investigated the impact of CSPAP programs on gross motor skills among students from low socioeconomic backgrounds (40, 46). Burns et al. (40) examined the effects of a 12-week CSPAP on gross motor skill development in 1,460 school-aged children (grades K-6) from low-income families, finding a 10% improvement in motor skills. Burns et al. (46) studied the effect of a year-long CSPAP on gross motor skills in 959 children (grades 1–6) from low-income schools, observing significant improvements. However, these studies lacked a control group, limiting its results’ validity, and also focused on the during-school components of CSPAP.

Furthermore, no identified research has examined the impact of after-school CSPAP component on students’ gross motor skills in children with low socioeconomic status. Additionally, no CSPAP studies have aimed to improve PMC-C and gross motor skills simultaneously. Therefore, this study aimed to address the limitations of previous studies and fill the research gap by examining the impact of a five-week after-school program on overhand throwing and perceived motor competence in students with low socioeconomic status.

Examining the impact of an after-school intervention on students with low socioeconomic status’ gross motor skills and perceived motor competence may help researchers implement interventions based on perceived motor competence and gross motor skills and design cost-effective interventions to help children with low socioeconomic status attain gross motor skills and increase their perception of motor competence.

Previous research has demonstrated the positive impact of CSPAP on improving students’ gross motor skills with students from low-socioeconomic backgrounds (40, 46). Additionally, Chan et al. (30) suggest that providing fun, structured, and supportive environments enhances students’ perceived competence. Therefore, we hypothesized that a five-week after-school program would effectively improve students’ overhand throwing skills and perceived competence.

## 2 Materials and methods

A quasi-experimental design was used to compare the gross motor skills of participants in the intervention and control groups. This study received approval from the University Institutional Review Board before data collection. Written assent was obtained from the students, and written consent was obtained from the parents after providing detailed information about the study's purpose, procedures, and potential risks.

### 2.1 Participants/setting

The study was conducted at a Title 1 school in Chandler, Arizona. A total of 80 elementary school students in grades kindergarten to 6th participated in this study. The intervention group consisted of 60 students (26 boys, 34 girls; *M*_grade_ = 4.27, *SD* = .43), while the control group consisted of 20 students (7 boys, 13 girls; *M*_grade_ = 4.00, *SD* = .35). The majority of the participants were Hispanic (86.3%), followed by African American/Black (6.3%) and Mixed (5.0).

Participants were recruited through parental consent and student assent forms sent to all students at the school. Only students who returned signed consent forms and agreed to participate were included in the study. No explicit inclusion or exclusion criteria were applied, as the goal was to maximize participation and include a diverse group representative of the school population.

### 2.2 Teacher

The first author delivered all the after-school program skill lessons, holds a bachelor's and a master's degree in Physical Education, has six years of experience teaching Physical Education, and is currently enrolled in a PhD program in Physical Education. Additionally, the first author received training in Dynamic Physical Education (DPE) curriculum (64) during his university studies and was trained on TGMD-2 by a member of the research team.

### 2.3 After-school program motor skill intervention

The students in the intervention group were taught 25 min once a week for 5 weeks. The first author taught the lessons using the Dynamic Physical Education (DPE) for Elementary School Children curriculum (64). The DPE curriculum is comprised of four parts: introduction, fitness, skill focus, and closing activity. However, given the brief duration in the after-school program (25 min), the research/school team adjusted the DPE curriculum by consolidating it into three sections, omitting the fitness component.

Consequently, each lesson began with a 5 min general warm-up, followed by the skill focus phase. In this phase, students were split into four groups, each rotating through four stations. The activities at these stations were designed to enhance students’ overhand throwing skills through engaging exercises. For example, students aimed at hitting targets on the wall with overhand throws, progressively increasing their distance to challenge their accuracy and strength. They also focused on stepping back while throwing to improve coordination and footwork. Additionally, they worked on knocking down cones using overhand throws, which encouraged precision and control in a fun, goal-oriented environment.

The students were provided five skill cues before starting the station activities (side to target, uppercase L, step-twist-throw, step with your opposite foot, follow throw) and were asked to perform overhand throwing without the ball before starting the activities at the stations. Each group stayed for 2–3 min at every station and then moved to the next station when they heard the whistle, as instructed.

In the closing part of the after-school program lesson, the students engaged in 10 min of gameplay emphasizing overhand throwing. This gameplay was designed to apply the skills learned in a dynamic and interactive setting, enhancing the students’ ability to transfer skills to real game situations. All the games were small-sided to ensure more engagement, and every student had a ball. An example of these games is knocking the cones. Here, the students were divided into four teams, with each team staying in one quadrant of the gym. Each quadrant of the gym had many cones, and each team tried to knock out the cones of the team in the quadrant opposite them with a ball by performing overhand throwing while defending their cones. The students were reminded to follow the four steps for successful overhand throwing during the game. The first author provided individual feedback to the students when needed.

To ensure fidelity to the curriculum, the first author followed a detailed lesson plan based on DPE curriculum for each session. These plans were reviewed by a faculty advisor with expertise in DPE prior to implementation. Additionally, two members of the research team, both with expertise in the DPE curriculum, observed each session to ensure adherence to the planned activities. Observations confirmed 100% implementation of the curriculum, and any necessary adjustments were addressed in real time to maintain consistency and alignment with the intervention structure. Feedback from the research team was provided to the first author throughout the study to further ensure fidelity to the curriculum.

### 2.4 Control group

The control group consisted of students who did not participate in the after-school program. With parental consent and student assent, these students followed their regular physical education curriculum. Outside of Physical Education, students in the control group were asked to follow their normal routines without additional structured physical activity. This contrasts with the CSPAP intervention, which provided targeted skill-based instruction in overhand throwing through a structured and supportive environment.

### 2.5 Instruments

#### 2.5.1 Overhand throwing

The study utilized the Test of Gross Motor Development-2 (TGMD-2) to assess overhand throwing, a skill chosen for its relevance across multiple sports and activities. TGMD-2 demonstrated strong reliability, with coefficients of 0.87 for content sampling, 0.98 for interscorer differences, and 0.88 for time sampling (47). Additionally, its validity was established through content description, criterion prediction, and construct identification, based on data collected from a sample of 1,208 children across 10 states in the U.S (47).

TGMD-2 consists of two subtests: locomotor (run, hop, leap, horizontal jump, slide) and object control (striking a stationary ball, stationary dribble, catch, kick, overhand throw, and underhand roll). The overhand subtest from TGMD-2 was only used to assess changes in the students’ overhand throwing following the intervention. The overhand throwing subtest consisted of four criteria. Each criterion was assessed with one point, that is, if the student performed it, they would get 1; otherwise, they would get 0. The first author followed the CDC (48) and TGMD-2 manuals for test administration (47). Two members of the research team administered and assessed the pre- and post-tests for both the control and intervention groups. Having previously achieved 100% agreement in scoring the overhand throw, they maintained this consistency throughout both assessments.

TGMD-2 was used due to its well-established reliability and validity in similar populations of elementary school children and its widespread use in assessing gross motor skills. The research team did not use the Throw-Catch Assessment, which assesses gross motor skills in a more authentic and dynamic environment, as this instrument was developed after the study was conducted.

#### 2.5.2 Perception of motor competence

The study utilized the Perceived Motor Competence in Childhood (PMC-C) questionnaire to assess the students’ perception of overhand throwing competence following the five-week after-school intervention. The PMC-C questionnaire consisted of 24-item Likert scale questions to assess the students’ self-perceptions of motor skills. Dreiskaemper et al. (49) validated the questionnaire, demonstrating its reliability (polychoric alpha coefficients ranging from .79 to .91) and validity through confirmatory factor analysis with good model fit indices for a similar student population. The students answered questions related to overhand throwing competency on a scale of 1 (strongly agree) to 5 (strongly disagree) pre-intervention and post-intervention.

### 2.6 Procedures

First, a pre-test was conducted for both the intervention and control groups. Upon entering the gymnasium, students started with a general warm-up and PA, supervised by a research team member. Another team member directed students to the testing area in the gym's corner, one at a time. The testing area was marked by a cone and a ball, positioned 20 feet from the wall. To minimize peer influence during the test, students faced away from the center while participating in PA.

Assessments were conducted individually. Initially, the researcher demonstrated an overhand throw. Subsequently, students were instructed to throw the ball as far as they could toward the wall without receiving specific instructions on technique (50).

Both PMC-C and TGMD-2 were subsequently administered to the students during pre- and post-testing. In order to maximize program intervention time, a team member administered the PMC-C survey during the school day with students by class. Students were asked to read the instructions and discuss a sample question. They were given ample time to complete the assessment.

On the final day, assessment procedures were replicated for the post-test for both groups. The same team member conducted both the p애re- post-tests.

### 2.7 Data analysis

To verify the assumption of normal distribution, a prerequisite for conducting an Analysis of Covariance (ANCOVA), the Kolmogorov-Smirnov test was performed. Then the ANCOVA was conducted to examine the difference in TGMD-2 and PMC-C test scores between groups, with pre-test scores and demographic variables included as covariates. Pre-test scores were included as covariates to control for baseline differences, to make sure that group comparisons accurately reflect the intervention's effect and enhancing the validity of the results.

Multiple regression analyses were performed to investigate the predictive value of changes in scores from pre-test to post-test for TGMD-2, and to determine if these changes predicted similar changes in PMC-C scores. Additionally, a separate regression analysis was performed to assess the impact of attendance on score changes from pre- to post-test for both TGMD-2 and PMC-C. The significance level was set at *p* < .05.

## 3 Results

The Kolmogorov-Smirnov test indicated that the data for both TGMD-2 and PMC-C post-test scores were appropriately distributed, thereby meeting the assumption of normality required for conducting ANCOVA. The model was statistically significant [*F*(4,57) = 6.14, *p* < .001, *η*_p_^2^ = .30], indicating that 30.1% of the variance in TGMD-2 post-test scores was explained by the covariates and independent variable (*R*^2^ = .30, *R*^2^_adj._ = .25). Group assignment (i.e., intervention vs. control) significantly predicted post-test scores [*F*(1,57) = 7.23, *p* = .01, *η*_p_^2^ = .11], showing a moderate effect size. Pre-test scores were also a significant predictor, [*F*(1,57) = 10.27, *p* < .01, *η*_p_^2^ = .15], with a moderate effect size. However, neither gender [*F*(1,57) = 0.34, *p* = .56, *η*_p_^2^ = .01 nor grade [*F*(1,57) = 3.04, *p* = .09, *η*_p_^2^ = .05], were significant predictors, both demonstrating small effect sizes. The mean and standard deviation of the test scores (i.e., TGMD-2, PMC-C) for both groups and the test statistics are detailed in Table 1.

**Table 1:** Means, standard deviations, and ANCOVA results for TGMD-II and PMC-C scores by group and test time (Pre-test and post-test).

	TGMD-II				PMC-C			
	Pre-test	Post-test	Δ	*F*	Pre-test	Post-test	Δ	*F*
Intervention	3.64 ± 1.37	5.23 ± 2.24	1.59 ± 2.31	–	9.84 ± 2.73	12.07 ± 2.13	8.34 ± 11.96	–
Control	3.17 ± 1.76	3.56 ± 2.06	.39 ± 1.46	–	10.0 ± 2.64	11.61 ± 2.79	3.17 ± 14.71	–
ANCOVA	–	–	–	6.14***	–	–	–	2.53*
Group assignment	–	–	–	7.23**	–	–	–	.62
*Covariate*								
Pre-test scores	–	–	–	10.27**	–	–	–	2.16
Gender	–	–	–	.34	–	–	–	2.56
Grade level	–	–	–	3.04	–	–	–	.46

Statistical significance: *p ≤ .05, **p ≤ .01, ***p ≤ .001

A separate ANCOVA was performed to assess the impact of group differences on post-test scores of the PMC-C test, with PMC-C pre-test scores and demographic variables serving as covariates. The overall model was statistically significant [*F*(4,57) = 2.53, *p* = .05], explaining 15.1% of the variance in PMC-C post-test scores (*R*^2^ = .15 *R*^2^_adj._ = .09). However, the main effect of group assignment (i.e., intervention vs. control) was not statistically significant, [*F*(1,57) = 0.62, *p* = .43, *η*_p_^2^ = .01], suggesting that there was no significant difference in post-intervention PMC-C between the control and intervention groups. Similarly, the main effects of gender and grade were non-significant, [*F*(1,57) = 2.56, *p* = .12 *η*_p_^2^ = .04] and [*F*(1,57) = 0.46, *p* = .50, *η*_p_^2^ < .01], respectively. The covariate, PMC-C pre-test scores, was also not statistically significant [*F*(1,57) = 2.16, *p* = .15, *η*_p_^2^=.04].

A regression analysis was conducted to examine whether the difference in scores from pre-test to pos*t*-test on the TGMD-2 predicted the difference in scores from pre-test to pos*t*-test on the PMC-C. The predictive value of the change in TGMD-2 scores was not statistically significant, with the regression equation not accounting for a significant proportion of variance in PMC-C score differences [*F*(1, 42) = 2.14, *p* = .15]. It was found that the differences in scores from pre-test to post-test on TGMD-2 did not significantly predict the differences in scores from pre- to pos*t*-test on PMC-C (*β* = .22, *p* = .15).

A separate regression analysis was performed to examine the predictive value of attendance on the differences in scores from pre-test to post-test on TGMD-2 and the PMC-C.

The results indicated that attendance was a significant predictor for the score differences in both TGMD-2 and PMC-C. Specifically, the regression model for TGMD-2 post-test score differences was significant [*F*(1, 42) = 5.54, *p* = .02]. Similarly, for PMC-C post-test score differences, the model was significant, [*F*(1, 42) = 7.72, *p* < .01]. It was found that attendance significantly predicted the differences in scores from pre- to post-test on TGMD-2 (*β* = .34, *p* = .02) and PMC-C (*β* = .44 *p* < .01).

## 4 Discussion

The study aimed to determine the effect of a 5-week CSPAP on students’ overhand throwing and perceived motor competence with students from low-socioeconomic backgrounds. Results from this study show that students in the CSPAP improved significantly from pre- to post program for the motor skill of overhand throw. The results of this study align with Burns et al. (40) and Burns et al. (40), who found that CSPAP significantly improved gross motor skills for students with low-socioeconomic status.

A significant difference in overhand throwing in favor of the intervention group was expected, given that after-school programs provide an opportunity to focus on children's motor-skill development beyond typical U.S. Physical Education programs, which emphasize fitness, sports, and games, as noted by Luz et al. (51).

The significant improvement in overhand throw skill development is noteworthy, considering the low level of gross motor skills observed before the intervention see Table 1. These findings affirm the effectiveness of CSPAP in enhancing gross motor skills among children with low socioeconomic status. Such enhancements are likely to result in greater PA engagement, higher health-related fitness, and enhanced cardio-metabolic health as these children progress into adolescence and adulthood (46).

Unlike other studies that observed age-related differences (46) and gender-specific variations in gross motor skills—with girls specifically outperforming boys in gross motor skills (52)—no significant differences were found in gender and age in this study. This outcome can be attributed to the nature of the intervention, where all activities were game-like, structured with stations, and each student had access to a ball. This setup limited opportunities for more skilled or older students to dominate the activities, thereby equalizing participation among all students.

The significant improvement in overhand throwing did not significantly improve perceived competence in the intervention group. This could be attributed to four reasons. First, Albaloul et al. (53) demonstrated that a sufficient intervention duration can significantly enhance children's perceived motor competence. In contrast, the short duration of the current intervention may not have provided students with enough time to fully recognize their motor skill improvements, thereby limiting its impact on their perceived competence. Second, focusing solely on gross motor skills might not effectively improve perceived motor competence. As Bandura (54) suggests, verbal persuasion is essential to enhance individuals’ belief in their abilities and skills. Third, PMC-C assesses perceived motor competence across eight distinct motor skills, not just overhand throwing. Since our intervention targeted only overhand throwing, it is unsurprising that overall perceived motor competence, encompassing all eight skills, remained unchanged. Improvements in a single skill may not substantially influence overall perceived motor competence. Fourth, younger children might not accurately perceive their actual motor competence due to limited cognitive development at their age (24, 29). However, it is interesting that previous studies found that younger children cannot accurately perceive their actual competence, unlike our study, they tend to overestimate their level. A possible explanation for this is that the previous studies were cross-sectional (55, 56), assessing students in a physical education setting, while in our study, children were assessed in an after-school intervention program. Schmidt and Lee (57) suggest that students assess their competency not merely by their performance and what they can accomplish. Given that the after-school program in this study, which included students from kindergarten to 6th grade, participants may have evaluated their achievements relative to their peers, affecting their perceived competence. This is because young children are more vulnerable to peer influence (58), which can result in a lower perception of their abilities (30).

### 4.1 Strengths

To the best of the authors’ knowledge, this is the first study that has explored the effects of the after-school CSPAP component on students’ gross motor skills in students with low socioeconomic status. Additionally, this is the first study to simultaneously examine the impact of CSPAP on both gross motor skills and perceived motor competence. Although the increase in perceived motor competence was non-significant, the study is crucial as it identifies potential variables that could be adjusted for future studies. Another strength of the study is the short duration of the intervention and each session, which was 25 min once a week. After-school programs are not intended solely to teach students motor skills or engage them in PA, as they typically include time for other activities like homework and snacks. Our study demonstrated that 25 min can effectively improve students’ overhand throwing.

### 4.2 Limitations

The study's results should be interpreted with caution; first, the study was conducted at a single Title I school, which limits the applicability of the findings to schools in different socioeconomic or geographic contexts. The unique characteristics of the participating school and community may not represent broader populations, and thus, the results should be interpreted with caution when applied to other settings.

Second, in our study, the control group had fewer students than the intervention class, which can decrease the reliability of the findings. Third, our study utilized a quasi-experimental design and employed convenience sampling, which may introduce selection and sampling biases, respectively. These factors limit the ability to generalize the findings to broader populations and may affect the validity of causal inferences regarding the intervention's effectiveness. Third, while the overhand throwing sub-test was selected due to the importance of overhand throwing and its association with many sports, utilizing all the skills included in TGMD-2 could give us a more comprehensive view of the impact of after-school programs on gross motor skills. Video recording is recognized for allowing multiple reviews and enhancing the reliability of assessments (30). However, this method was not utilized because parental approval for video recording was not granted, which may have impacted the precision of our observations and overall findings.

## 5 Conclusion

The findings from this study suggest that even a short 5-week afterschool program, meeting just once a week, can significantly improve students’ overhand throwing skills, demonstrating the effectiveness of brief, targeted interventions in enhancing motor performance.

Future studies should consider a longer intervention period with approximately equal sizes for intervention and control groups. Future research should also use tools to assess overhand throwing more authentically, such as TGMD-2, which, while widely used, assesses gross motor skills in an isolated environment. Future studies could specifically utilize the Throw-Catch Assessment (TCA) instrument, which was recently developed to assess gross motor skills in an authentic and dynamic environment (59). Future studies should also explore methods to enhance students’ perceived motor competence within the context of after-school programs. Specifically, interventions could adopt strategies suggested by Gao et al. (60), which emphasize providing concurrent feedback from both teachers and peers during activities to bolster students’ perceived motor competence in their motor skills. Finally, future studies using different curricula, such as Sport Education (61), which has been shown to improve perceived motor competence (53), may be useful in improving students’ perceptions of their motor competence.

In addition, policymakers could support initiatives that allocate funding and resources to implement evidence-based motor skill interventions within after-school settings. By prioritizing programs that foster both skill development and perceived motor competence, after-school programs could play a vital role in promoting long-term physical activity engagement and overall child development.

## Data Availability

The raw data supporting the conclusions of this article will be made available by the authors, without undue reservation.
